# Occupancy Prediction Using Low-Cost and Low-Resolution Heat Sensors for Smart Offices

**DOI:** 10.3390/s20195497

**Published:** 2020-09-25

**Authors:** Beril Sirmacek, Maria Riveiro

**Affiliations:** Jönköping AI Lab (JAIL), Department of Computer Science and Informatics, School of Engineering, Jönköping University, 553 18 Jönköping, Sweden; maria.riveiro@ju.se

**Keywords:** heat sensors, smart offices, occupancy prediction, machine learning, computer vision, feature engineering, explainability, explainable AI

## Abstract

Solving the challenge of occupancy prediction is crucial in order to design efficient and sustainable office spaces and automate lighting, heating, and air circulation in these facilities. In office spaces where large areas need to be observed, multiple sensors must be used for full coverage. In these cases, it is normally important to keep the costs low, but also to make sure that the privacy of the people who use such environments are preserved. Low-cost and low-resolution heat (thermal) sensors can be very useful to build solutions that address these concerns. However, they are extremely sensitive to noise artifacts which might be caused by heat prints of the people who left the space or by other objects, which are either using electricity or exposed to sunlight. There are some earlier solutions for occupancy prediction that employ low-resolution heat sensors; however, they have not addressed nor compensated for such heat artifacts. Therefore, in this paper, we presented a low-cost and low-energy consuming smart space implementation to predict the number of people in the environment based on whether their activity is static or dynamic in time. We used a low-resolution (8×8) and non-intrusive heat sensor to collect data from an actual meeting room. We proposed two novel workflows to predict the occupancy; one that is based on computer vision and one based on machine learning. Besides comparing the advantages and disadvantages of these different workflows, we used several state-of-the-art explainability methods in order to provide a detailed analysis of the algorithm parameters and how the image properties influence the resulting performance. Furthermore, we analyzed noise resources that affect the heat sensor data. The experiments show that the feature classification based method gives high accuracy when the data are clean from noise artifacts. However, when there are noise artifacts, the computer vision based method can compensate for those artifacts providing robust results. Because the computer vision based method requires an empty room recording, the feature classification based method should be chosen either when there is no expectancy of seeing noise artifacts in the data or when there is no empty recording available. We hope that our analysis brings light into understanding how to handle very low-resolution heat images in these environments. The presented workflows could be used in various domains and applications other than smart offices, where occupancy prediction is essential, e.g., for elderly care.

## 1. Introduction

Sensors and electronic devices become increasingly integrated in our environment and daily routines. Because of the advancements in these technologies, their continuous decreased costs (even edge devices) and ease of deployment (using sensor fusion, cloud, and Internet of Things—IoT) the design of smart environments have gained a lot of attention in recent years. Using sensor technologies combined with small and efficient hardware and intelligent algorithms, we are now able to provide smart solutions that support humans at home and work, e.g., in business offices, school buildings, hospitals, elderly facilities, and wellness centers. Smart environments differ from traditional environments, because of the real-time interactions between the users and the facilities. In this context, two important research topics are occupancy prediction and human activity detection and recognition. While activity recognition normally refers to identifying movements and actions from sensor data, predicting room occupancy tackles the problem of estimating the number of people in a given room or space. In this study, we focus on the detection of people occupying a certain area—a meeting room, developing occupancy prediction solutions.

In general, detecting human activities accurately and in real-time is a challenging problem for several reasons [1]. There are several methods developed to detect human activity indoors, e.g., using multimedia-sources (such as audio/video) [2,3], wearable devices (smartwatches, wristbands, etc.) [4], and ambient sensing [5]. Each method comes with its advantages and disadvantages that are based on its use cases. For instance, despite having high accuracy, audio/video-based solutions bring challenges in real implementations because of the privacy regulations. Similarly, wearable device-based methods can provide customized solutions, but they might also lead to discomfort issues, negatively affecting their feasibility. Ambient sensing (such as infrared sensors), on the other hand, relies on sensors that provide only ambient information about the environment. It does not have any privacy or discomfort issues, but it requires careful thinking in terms of sensor placement and development of intelligent solutions that can extract information from sensor data that does not provide sharp visual features to understand the environment easily. Ambient sensing systems that are generally used for detecting human activities install a large number of sensors (same or of different modality) in the environment. Each sensor brings sensing noise and such noise needs to be handled before the output is fed to a sensor data fusion module, a decision-making system or a machine learning model.

Smart spaces are usually shared by multiple people. In order to automate the facilities, i.e., heating, ventilation, air conditioning, it is important to know how many people are there, at which location, and what do they do [6,7]. When smart offices are considered, this information is also important to address safety issues and an efficient meeting room occupancy management. Real-time human activity detection and prediction can be used to effectively allocate resources in order to increase user comfort in conference rooms in offices [8] as well as study-rooms in households [9]. Robotics systems can also greatly improve the success of such automation units [10].

Existing solutions for occupancy prediction often require higher resolution sensors that are expensive and raise privacy issues that are related to the people who use the environment (see background section for more details). In turn, solutions that make use of lower resolution sensors have not been widely tested and their performances are mostly illustrated within controlled environments, where there is no sun exposure difference during the day, heat sources from other objects, or heat prints left from people who used the chairs and desks for a long time. Therefore, a deeper analysis of the effects of this noise is necessary in order to deploy accurate solutions in real environments. We believe that low-cost and low-resolution heat sensors would be helpful for creating easily-accessible solutions for many smart offices while avoiding privacy concerns of the people who use them (it is impossible to identify the users in the environment in these low-resolution images). However, one of their major challenges is the high sensitivity to other heat sources; this must be considered in order to provide robust algorithms that can adapt to the changing conditions of the environment. Thus, in this paper, we explore how to use low-cost and low-resolution heat sensors that are looking for solutions that are robust to noise artifacts. We present two approaches, one computer vision-based, and the other one, machine learning-based. We evaluate and compare their performance with data that were recorded in a real office environment, and we discuss their advantages and disadvantages, as well as their particularities regarding different heat artifacts.

In particular, this paper provides the following contributions:

1. We propose two different workflows (one based on computer vision and one based on machine learning) for predicting the number of people in the environment.
2. We evaluate our novel workflows and provide an exhaustive analysis of their performances in a real office environment, where several meetings happen while the temperature and the sun illumination from the windows change during the day.
3. We explain the relation of the algorithm parameters to the installation position of the sensor in the office environment.
4. We use explainable AI methods in order to investigate which algorithm parameters and image properties provide useful information to the classifications.
5. Finally, we focus on understanding the heat artifacts in recordings that are acquired from an environment that is heavily exposed to the sunlight and used by many people in long meetings. We discuss the different effects of the environmental heat artifacts (like sunlight) and the heat prints of the people who sat on a specific location for a long time. We discuss the capabilities of our workflows to deal with such noise artifacts and we offer compensation methods in order to increase the accuracy of the results.

## 2. Background

The human activity detection problem is solved in different ways in the literature. Activity detection has usually been performed while using audio/video surveillance, because multimedia data provide very accurate representations of human activities [11]. Another method for obtaining activity detection is using wearable devices [12]. This method has gained a lot of attention due to wearable devices becoming widely-available and popular. Nevertheless, limitations of wearable device-based applications are obvious. First of all, people might forget or not cooperate with the sensors that they are wearing. Secondly, new people visiting the environment cannot be tracked when they do not have sensors.

A lot of companies and researchers turned their focus on using low-cost and low-resolution sensors for keeping the prices low and keeping people’s privacy in the scene due to the high prices and the privacy issues raised by surveillance cameras. Passive infrared (PIR) and heat sensors became interesting for such applications and researchers have focused on developing algorithms that can extract information, even when the data cannot obviously tell all important visual properties of the scene.

Wahl et al. [13] used PIR sensors to recognize activities, and to keep track of how many people were in a room. Murao et al. [14] used PIR sensors that are distributed in a house, in order to track the activation patterns of the sensors. They used these patterns to recognize activities and keep track of how many people were there. Even though PIR sensors are very low-cost, available to many researchers and they keep the privacy of the people involved, when they are used in a constrained area like a meeting room, the sensors need to be placed on all areas or objects of interest, as presented in [15]. Such an application requires many sensors, even for one room, at all locations where they could interfere with the activity being performed. Multiple devices also means that installation, maintenance, sensor fusion, etc. require more effort, even if the sensors are ”tape and forget”, as argued in [15]. Besides, even though one PIR sensor is much cheaper than one heat sensor, when so many of them are necessary in one room, the whole application might cost even more than a heat sensor alternative.

Heat sensors (infra-red sensors/cameras) provide heat images that can be conveniently used to track people, as they only can visualize heat reflections. Heat sensors can be used to detect and track people [16,17]. While higher resolution sensors provide more chances to algorithms in order to provide robust solutions, the prices of the heat sensors increase exponentially with respect to their increasing resolutions. Therefore, it is important to explore the possibilities of developing algorithms that can make some predictions relying on information that was extracted from images with very low resolution in order to keep the application low-cost.

Some researchers looked for opportunities for using extremely low-resolution heat sensors for understanding human activity indoors. Jeong et al. [18] showed that an adaptive thresholding method could help to detect people while using low-resolution heat sensors. Singh et al. [19] used adaptive thresholding and the binary results for understanding whether people are on the floor, in a standing or sitting position. Next, they extended their work towards a multiple sensor application [20]. Troost [21] compared different classifiers that are trained to predict the number of people in low-resolution heat sensor images. Gonzalez et al. [22] used a single sensor and trained a classifier to differentiate 21 different activities in the environment. The experiments gave successful results to show that it is possible to rely on such low-resolution image information to understand the usage of the environment. Johansson and Sandberg [23] looked for opportunities of training neural networks, specifically CNNs (Convolutional Neural Network) and LSTMs (Long Short-Term Memory), in order to have a mathematical model that can make predictions regarding the number of people visible in a low-resolution heat sensor. The experiments are restricted to data sets that are acquired in controlled environments (such as places that are not exposed to changing sunshine conditions from large windows—which are common in office spaces), as we see in the literature related to the usage of very low-resolution heat sensors. Robustness in changing environmental conditions and the generalization of the different algorithms tested have not been discussed either and, moreover, the research on thermal noise and compensation methods is still quite limited. Thus, in this study, we focus on these aspects, hoping to further understand how to solve them.

## 3. Aim and Objectives

Given the challenges of the studies described in the previous section, the aim of this work is to explore low-cost and privacy-preserving solutions for occupancy prediction that are robust and accurate and take the noise artifacts of the environment into account. After exploring possible solutions that fulfilled these criteria, we suggested two alternative workflows that are based on two different methods. One method heavily relies on the use of computer vision algorithms, while the second method relies on feature engineering (finding good/representative features in data) and solving the occupancy prediction problem through machine learning. When considering these two different workflows that we present in this work, we herein tackle the following three research questions:

- Can we rely on computer vision or machine learning based methods in order to provide robust occupancy prediction solutions when we use a low-cost and low-resolution sensor?
- What is the best occupancy prediction accuracy that we can achieve when computer vision-based or machine learning based methods are used?
- What are the sources of the noise artifacts and what are the possible noise compensation methods to overcome errors caused by them?

## 4. Scenario and Data

**Figure 1:**
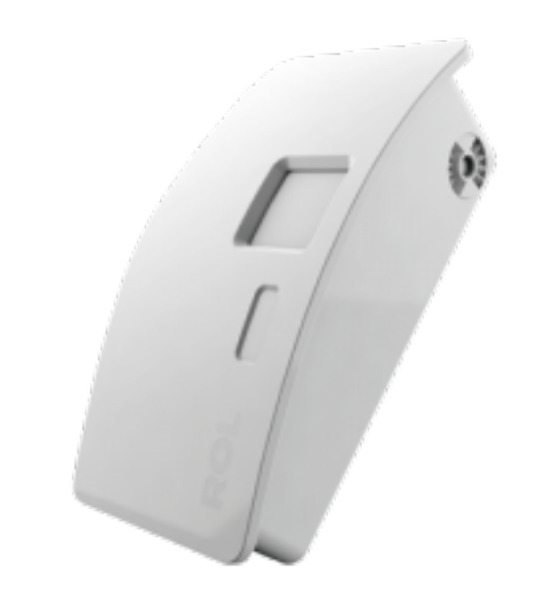
The low-cost and low-resolution heat sensor used in this study [24].

**Figure 2:**
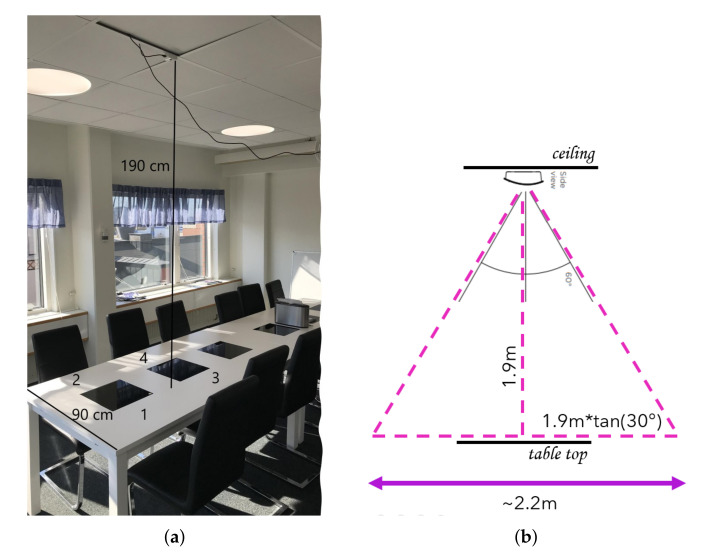
(**a**) The meeting room where the test data streams were collected. The sensor was placed at the ceiling, 1.9 m above the table. The seat numbers correspond to the person presence numbers in Table 1. (**b**) Field of view calculation for the meeting room setup. Table top line corresponds to the black line at the table edge with 90 cm measurement in (**a**).

Figure 1 and Figure 2 present the low-cost low-resolution heat sensor used in this study [24], as well as the environment used to collect the data. The sensor provides 8×8 frame size images that show the temperature in the field of view as degree Celsius using a grayscale value for each pixel. The sensor can capture 10 raw heat frames per second. The sensor description is provided by the manufacturer at [25]. Table 1 shows the heat recording data set which we have used for testing our algorithms. The data set was collected in three different dates while using the same sensor that was located at the office ceiling, as shown in Figure 2. In these three different meeting days, the meeting room was used by people that worked at the smart office company who randomly chose their seats. The figure also depicts the labels for the chair ID’s, which we have indicated whether there is a person or not in the data set description part of Table 1. In the table, the blue highlighted rows correspond to the sensor recordings that are captured when the room is empty. Later, we will use these recordings for noise analysis and noise compensation. For the performance analysis of workflow 1 and workflow 2, we have used the Day 1 and Day 2 data set. We have used the 24 h recording of the Day 3 data for the assessment of the explainability methods, since the recording includes significant environment temperature changes.

## 5. Methods

In this section, we introduce two different workflows in order to determine the number of people in each frame (image) of the heat sensor recordings. The first method uses traditional computer vision algorithms (aka computer vision based occupancy prediction), while the second one is a machine learning-based solution. The machine learning based approach creates a feature vector for each frame and, after that, a supervised classification algorithm learns how to fit an optimal model in order to be able to classify feature vectors for identifying the number of people in the scene (we refer to this solution as feature classification based occupancy prediction). In the remainder of this section, we focus on the theory of the modules that construct our workflows, and we provide a detailed analysis with the different data sets in the Experimental Results section.

### 5.1. Computer Vision Based Occupancy Prediction

**Figure 3:**
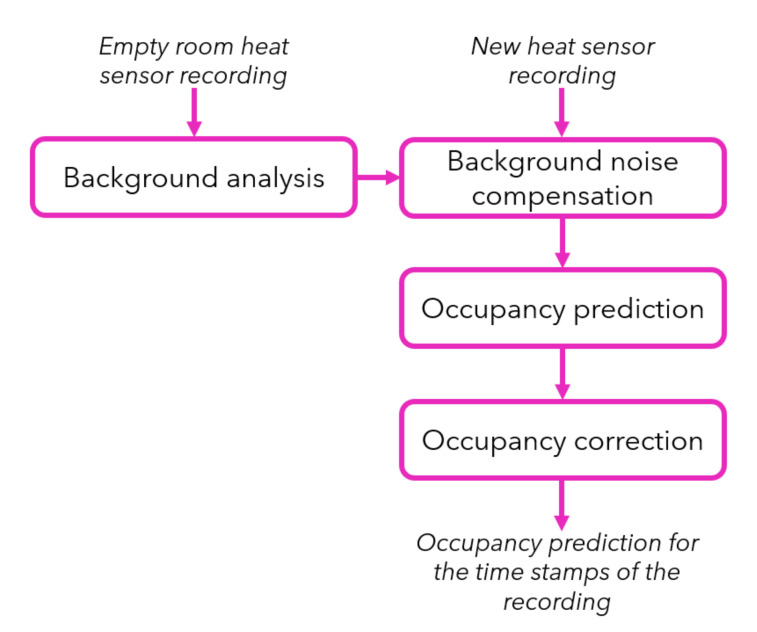
Workflow of the computer vision based occupancy prediction method.

Figure 3 presents the workflow of the computer vision based occupancy prediction approach. This solution requires a baseline, i.e., a recording when the office is empty. Each module of the workflow is described in detail hereafter.

#### 5.1.1. Background Analysis

We assume that we can improve the quality of each frame by removing the noise effects coming from other heated objects in the environment, such as computer-like devices or other objects that are exposed to sunlight coming from windows. We assume that we can use a short recording of the empty room before any meeting for compensation of such heat artifacts. To do so, we calculate the mean and standard deviation of each pixel in an empty room recording with our background analysis Algorithm 1. Thus, the background analysis module provides the mean (m(x,y)) and standard deviation (s(x,y)) of each pixel value through the recording of the empty room. Later on, when a new heat sensor recording is captured, these two matrices are used for background noise compensation.
**Algorithm 1:** Background analysis algorithm
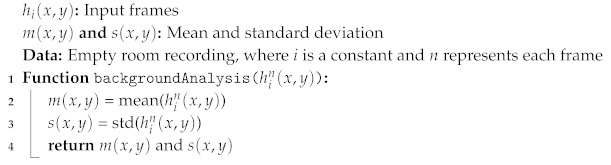


**Figure 4:**
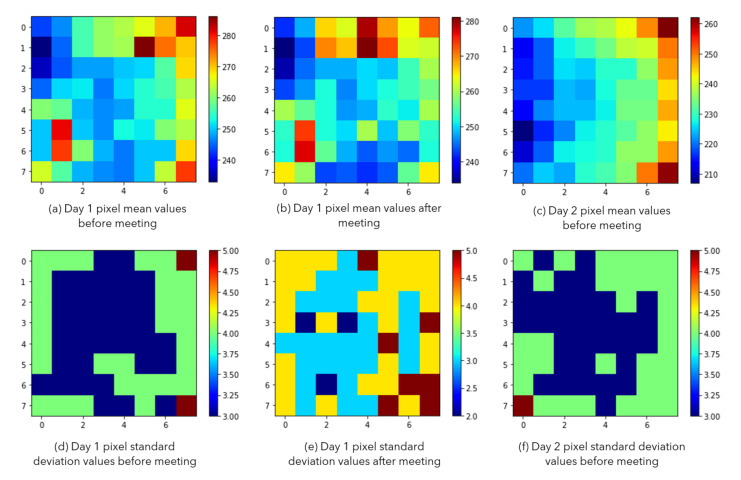
(**a**,**b**) show the pixel mean values (m(x,y)) for the empty room recording in Day 1 data ($h_{1}\left(x,y\right)$ and $h_{2}\left(x,y\right)$) before and after meeting, (**c**) shows the pixel mean values for the empty room recording in Day 2 data ($h_{10}\left(x,y\right)$) before the meeting, (**d**,**e**) show the pixel standard deviation values (s(x,y)) for Day 1 data ($h_{1}\left(x,y\right)$ and $h_{2}\left(x,y\right)$) before and after meeting, (**f**) shows the pixel standard deviation values for ($h_{10}\left(x,y\right)$).

The blue highlighted rows presented in Table 1 indicate the recordings that are considered for doing the background analysis. Figure 4 shows the empty room mean and standard deviation based on those three recordings ($h_{1}\left(x,y\right)$, $h_{2}\left(x,y\right)$, and $h_{10}\left(x,y\right)$).

As seen in Figure 4, there are two empty office recordings in Day 1 and one empty room recording in Day 2. The recordings of Day 1 are taken before and after a meeting that took place in the office. The Day 2 recording is taken only before the meeting. When we compare the before meeting recordings of two different days (a and c), we notice that the mean values are different. We understand that, even though there are no heat prints from people yet, the environment is heated differently because of the sunlight exposure from large windows. Day 2 looks sunnier and warmer. Standard deviations of the frames for those recordings (d and f) look similar. However, even though the sunlight exposure did not change during the recordings of Day 1, before and after meeting recordings show some differences. We see a slight increase in the mean values (a and b). Besides, the standard deviations look higher for the after meeting recording (d and e are compared). We believe that the difference is caused by the heat prints that were left on the seats and the table edges where people were located.

#### 5.1.2. Background Noise Compensation

We use s(x,y), as calculated in the previous step, for removing the pixels that we cannot trust. For instance, if the heat fluctuation (standard deviation) of a pixel is larger than a pre-determined threshold value, then we do not use that pixel in the further process (we simply change the corresponding pixel values to zero in the occupied room recordings). If all pixels of s(x,y) have values that are lower than the threshold temperature, then s(x,y) matrix has no impact on the further processing steps. In our studies, we have chosen the heat threshold as 50 (corresponding to 5 Celsius degrees), since we have noticed that a human heat print causes more than 50 standard deviations when compared to the standard deviation of the empty office. This is the only fixed threshold value that we have selected by looking at the human heat prints in our data set. We have not noticed more than 5 Celsius degrees standard deviation when a person leaves a seat. However, this value could be related to the materials that are present in the chairs used. A different seat material could absorb more or less heat from the human body; therefore, it might be possible that this threshold value varies with the chair materials and compositions. For simplicity, we leave the detailed analysis of the impact of different seat materials to future works.

For removing the background noise, we have conducted experiments by employing the Fine Grained Saliency approach [26]. This method creates real-time saliency maps that indicate the image regions which look different than their surroundings. With this method, we expected to extract the heat resources which might indicate both people and also noisy pixels. Even though this saliency method gives very successful results on high resolution images, it did not perform well on our low-resolution data. Therefore, we believe that the noise compensation method that we proposed above is a reliable solution for this case and, in general, when low resolution images are used.

**Figure 5:**
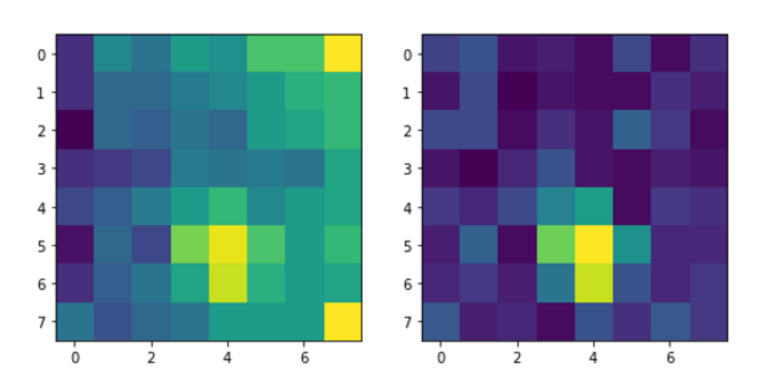
Left; the first frame of the $h_{13}\left(x,y\right)$ recording as an example of raw heat image having one person in the scene. Right; the same frame after applying the background noise removal technique.

We provide an example heat image frame before and after the background noise compensation in Figure 5. There is one person in this data; however, the original recording had very high heat levels on the right top and bottom pixels. The difference clearly shows that the background noise compensation algorithm can eliminate the noisy pixels and highlight the person in the scene.

#### 5.1.3. Occupancy Prediction

After the background noise compensation is applied, we use adaptive filtering and create a binary image from each frame. Therefore, we use the adaptive filtering method, which is also known as Otsu’s thresholding method [27]. This method automatically selects a threshold value by looking at the grey level histogram of the input images. After obtaining the binary image, we apply a bounding box around each binary segment.

The field of view of the sensor is 60 degrees in both vertical and horizontal directions [24]. In Figure 2b, we approximate the coverage area of each heat sensor frame as a side view. When considering the height of the sensor, we expect 2.2×2.2 m${}^{2}$ coverage area in the table top level. This means that, when we divide the area into 8×8, each pixel shows approx. an area of 0.27×0.27 m${}^{2}$. Assuming that one person covers between 0.5×0.5 m${}^{2}$ to 0.75×0.75 m${}^{2}$ (top view), we expect one person to cover from 2 to 3 pixels width in the image when a bounding box is located on the detected region.

Based on this assumption, when a bounding box width or height is larger than 3 pixels, we count the number of people by dividing the (width × height) to 3×3 and rounding the result to make it an integer value. In this way, we count the number of people in each frame. This predicted value can be presented real-time. However, if a correction is needed (because of any heat noise fluctuation during the recording), then the following correction module offers filtering and produces a post-processed result.

#### 5.1.4. Occupancy Correction

We post-process the results for correcting possible miscalculated frames after the real-time computations of the predictions are done. We filter the predicted values using a 5 frame size median filter after we extracted the number of people in each frame of the whole recording. This means that, if the predicted number of people in the *i*th frame is $N_{i}$, the filter considers $\left[N_{i-2},N_{i-1},N_{i},N_{i+1},N_{i+2}\right]$ for i≥2 for applying the median filter. Of course, this parameter should be carefully chosen, since it will affect the detection performance. If the frame size is small, e.g., 3, the filtering method would not be able to remove the noisy results that appear more than three times in sequential frames. If the frame size is large, then there are more chances to remove the noisy results that appear in sequential frames; however, the larger filter size can also remove true detection results when the number of people really changes in some frames for a very short time period. Therefore, this trade-off should be considered when tuning this parameter, depending on the expected noise and the expected human movement in the recordings.

### 5.2. Feature Classification Based Occupancy Prediction

**Figure 6:**
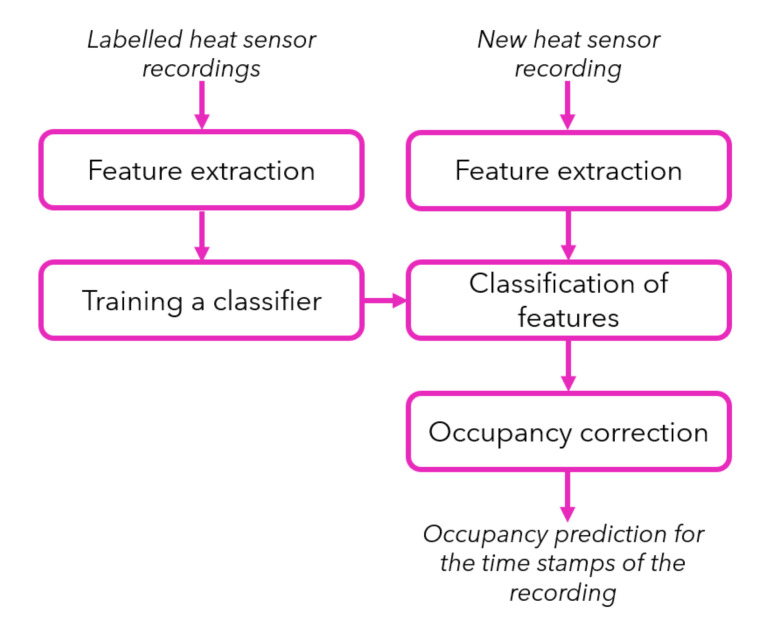
Workflow of the feature classification based occupancy prediction method.

The workflow of the second approach for occupancy prediction is presented in Figure 6. In contrast to the previous approach, we do not need a baseline image that represents an empty room, but we need labelled heat sensor recordings in order to train our classifier. With a reasonably good amount of labelled sensor recordings as example, we could train the classifier only once to use it new sensor data later on. With “labelled heat sensor recordings”, we mean that we need to know how many people are seen in the scene for each frame. It is not necessary to label the pixels of the frames in order to show where these people are located, since we are not concerned with the locations of the people in the scene. Nevertheless, we would like to extract representative features and train our classifier, so that it can identify the number of people in new frames. In the following sections, we describe each module of the workflow in detail.

#### 5.2.1. Feature Extraction

**Figure 7:**
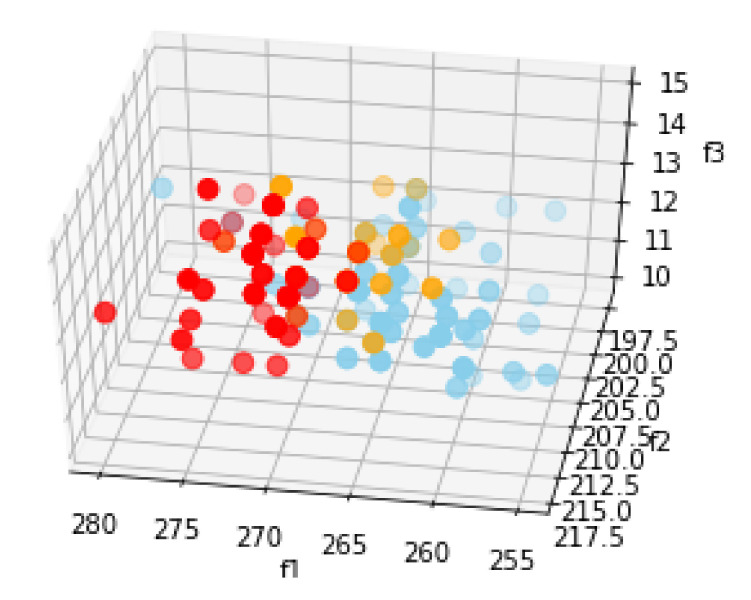
Training features extracted from heat sensor images of the office space when it is in three different states. Blue data points: features of the empty office recordings, orange data points: features of the office recordings when it is occupied by one person, red data points: features of the office recordings when it is occupied by two people.

For each *i*th frame of a $h^{i}\left(x,y\right)$ heat recording, a feature vector is constructed as $f_{i}=\left[\sigma_{i},\mu_{i},min\left(h^{i}\left(x,y\right)\right),max\left(h^{i}\left(x,y\right)\right)\right]$. Here, $\sigma_{i}$ and $\mu_{i}$ correspond to the standard deviation and mean values of all pixel within the *i*th frame called $h^{i}\left(x,y\right)$. $min(h^{i}\left(x,y\right))$ and $max(h^{i}\left(x,y\right))$ correspond to the smallest and the largest pixel values within the *i*th frame. Naming the four components of the $f_{i}$ feature vector as $\left[f_{0},f_{1},f_{2},f_{3}\right]$, we have plotted the $\left[f_{1},f_{2},f_{3}\right]$ feature values for the Day 2 recordings in Figure 7. Becasue we can only visualize three feature components at a time without applying feature reduction, we chose to visualize all but $f_{0}$; as compared to the scales of the other feature components, $f_{0}$ varies in a very small scale, which means that it is hard to visualize how the differences contribute to differentiate classes.

Three components of the Day 2 recording features in Figure 7 show that the feature space could be learned by a classifier in order to separate different classes showing different occupancy levels of the office.

#### 5.2.2. Training a Classifier

We use CatBoost classifier in order to classify $f_{i}$ feature vectors. CatBoost is a recently open-sourced machine learning algorithm [28]. It is able to work with diverse data types to help solving a wide range of problems that data scientists face today. “Boost” comes from gradient boosting machine learning algorithm, as this library is based on a gradient boosting library. Gradient boosting is a powerful machine learning algorithm that is widely applied to multiple types of business challenges, like fraud detection, recommendation systems, forecasting, etc. It can return very good results with relatively little data, unlike deep learning models that need to learn from massive amounts of data. Moreover, CatBoost works well with multiple categories of data, such as audio, text, images, and historical data.

CatBoost is especially powerful in two ways:

1. it yields state of the art results without extensive data training typically required by other machine learning methods, and
2. it provides powerful out of the box support for the more descriptive data formats that are associated with many data science problems. We used the CatBoost library [29] and trained a classifier, which learns how to distinguish the feature vectors when they are labelled with the number of people in the room. As seen in the workflow from Figure 6, the training is only performed once and the trained classifier is used to classify feature vectors when a new heat recording is processed.

#### 5.2.3. Classification of Features

When a new heat recording is captured, for each frame $f_{i}$ a feature vector is extracted and the trained classifier is used to identify in which class the feature vector falls. The classification result indicates the number of people in the room for each frame. The classifier can be used in real-time applications because the CatBoost classifier performs at an excellent computational speed, even for classifications of large feature vectors.

#### 5.2.4. Occupancy Correction

The occupancy correction module can be used as a post-processing step in order to eliminate the noisy computations in frames, as seen previously. Occupancy correction is done with the same median filtering method introduced in the previous Section 5.1.4 within the first workflow.

Hereafter, we introduce the results that were obtained after applying the two workflows previously described.

## 6. Experimental Results

In this section, we present the results of the performances of the two suggested workflows for occupancy prediction, discussing different use cases and their advantages and disadvantages. We use explainability methods in order to analyze the feature classification method of the second workflow in-depth.

We designed an experiment using data sets that were collected in three different days at the same ceiling position of the smart office in order to provide quantitative evaluation and comparison of our two workflows. The first and the second day data sets were recorded just before, during and after a meeting happened. In both two days, during the meeting, the total number of people changed (new people came and some people left the office). The third day data set also includes a meeting where the total number of people changed likewise. However, the third day data set was collected for 24 h uninterruptedly. Therefore, the third data set includes more information in order to analyze the correlation between the effects of the outdoor temperature to the indoor heat-sensor noise and quality factors. In the following subsections, we explain the quantitative assessment methods, results, and our reflections on them.

### 6.1. Analysis of the Computer Vision Based Method

We start with the performance analysis of the first workflow. Table 2 shows the performance of the computer vision based workflow for each heat recording used for testing. Here, TD stands for ’True Detection’, which corresponds to the percentage of people who are detected correctly in an overall recording. FP stands for ’False Positives’ and FN stands for ’False Negatives’. TD, FP, and FN values are calculated with the following equations; Total stands for the total number of people in the scene; TD=100×(Numberofpersonscorrectlydetected)/(Total), FP=100×(Numberofpersonsdetectedatincorrectlocations)/(Total) and FN=100×(Numberofpersonswhichcouldnotbedetected)/(Total). These definitions also mean that the sum of TD and FN values for a recording is always equal to 100. However, the FP value might be larger than 100% when there are more false detections than the total number of people within the scene.

**Figure 8:**
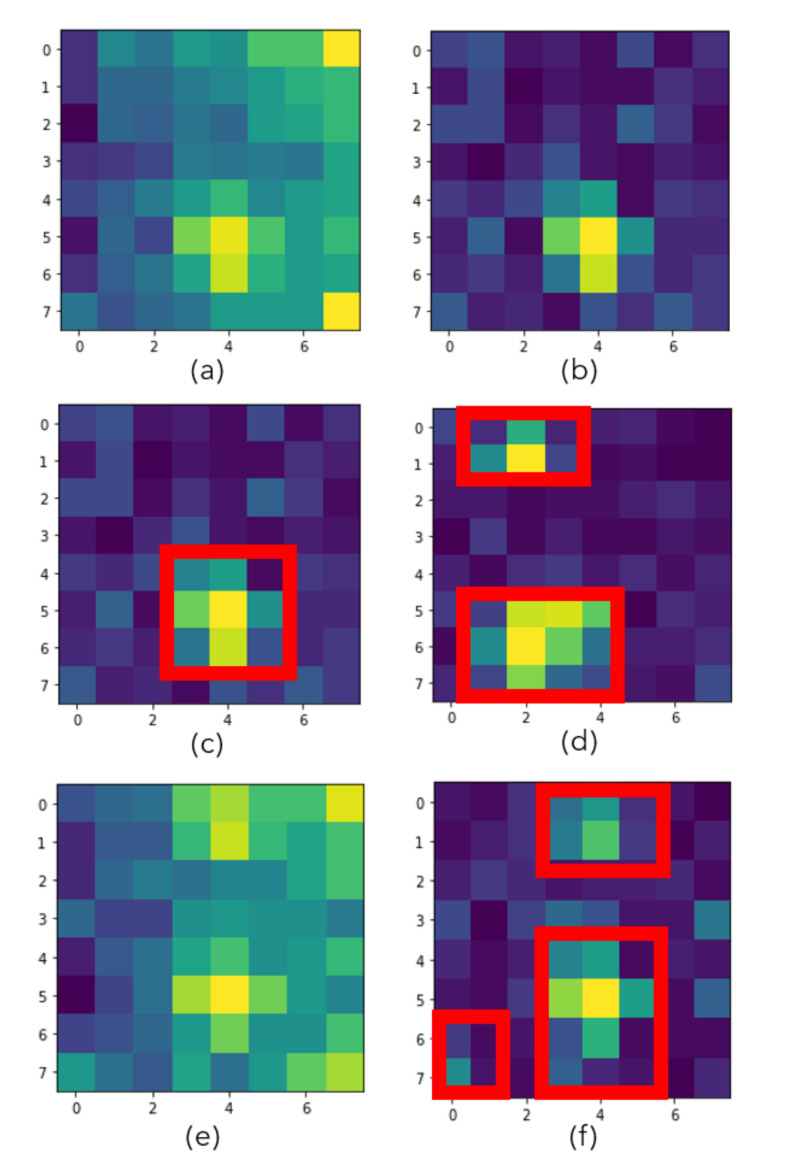
Analysis of the computer vision based method (workflow 1). (**a**) A raw image frame from $h_{13}\left(x,y\right)$ recording having one person in the scene. (**b**) Frame given in (**a**), after background noise compensation. (**c**) Occupancy detection result for the frame given in (**a**). (**d**) Occupancy prediction result of a frame from the $h_{12}\left(x,y\right)$ recording which has two people in the scene. (**e**) A raw frame from the $h_{14}\left(x,y\right)$ recording which again has two people in the scene but at different seats. (**f**) Occupancy prediction result of the frame given in (**e**), which, unfortunately, has a false positive due to the heated seat where a person was sitting earlier.

We noticed that the TD rate is 100% and the false detection rates are almost 0% in most of the Day 2 recordings. That is probably because of the low environmental noise artifacts (less outside heat and sun rays) during this day. In Day 2 recordings, only $h_{14}\left(x,y\right)$ is processed with a lower performance. When we visualize the detection results for this data, we see that a human heat print (because of a person who changed the seat) causes a false detection, see Figure 8f.

### 6.2. Analysis of the Feature Classification Based Method

The CatBoost classifier has been trained on the 70% randomly chosen frames of the Day 2 data set, the remainder 30% of the frames were used for testing. We obtained 97.64% classification performance (TD) with those frames. Because Day 2 data were captured with less sun exposure (as it was reported by the data collecting scientists), we have chosen Day 2 data in order to start and see the performance of our classifier on the data that we expect to find less noise. After seeing that the classifier performed good, we have applied the same experiment on the Day 1 data. We re-trained the classifier (from scratch) on the 70% randomly chosen frames of the Day 1 data set. We used the remainder 30% of the frames for testing. We achieved 95.48% performance. This result showed us that the classifier can be trained and give robust results, even in the presence of high noise artifacts.

Next, we have conducted experiments by using the classifier that was trained on the Day 1 data for making occupancy predictions on the Day 2 data. In this case, we have achieved 94.70% performance when considering the true detection percentage on the Day 2 data. We achieved 96.70% true detection performance on the Day 1 data when we mixed Day 1 and Day 2 data for training the classifier.

For giving a clearer view for discussion, we have tabulated the training sets, test sets, and the performances in Table 3. The experiments show a high performance of the feature based occupancy prediction method, even when it trained on a different day with different environmental conditions and used on another day again. However, if one classifier is going to be used in an environment that has significantly changing noise artifacts, the classifier will perform better when data from the different times of the day (or different days) are fused to train the classifier. Even though the classification performances are high in terms of TD percentages, we notice that the false negatives are high in Day 1 data, which include more environmental and human heat print caused noise. This is mainly because of that the feature classification based algorithm cannot do background noise compensation since the empty room recordings are not used for noise removal, as it was done in the computer vision based method.

### 6.3. Analysis of the Feature Contributions

As mentioned earlier, the feature vector is constructed while using four properties that we extract from each frame. However, it is possible that some of these properties make a very poor contribution to the overall classification. Therefore, we borrow methods from the recent eXplainable Artificial Intelligence (XAI) [30] field of research in order to further investigate the influence and effect of these proprieties. We use the well-known approach SHapley Additive exPlanations (SHAP) [31] in order to determine which properties of the frames are really effective in the classification module. The goal of SHAP is to explain the prediction of an instance *x* by computing the contribution of each feature to the prediction. The SHAP explanation method computes Shapley values from coalitional game theory. The feature values of a data instance act as players in a coalition; the Shapley values tell us how to fairly distribute the “payout” (= the prediction) among the features.

**Figure 9:**
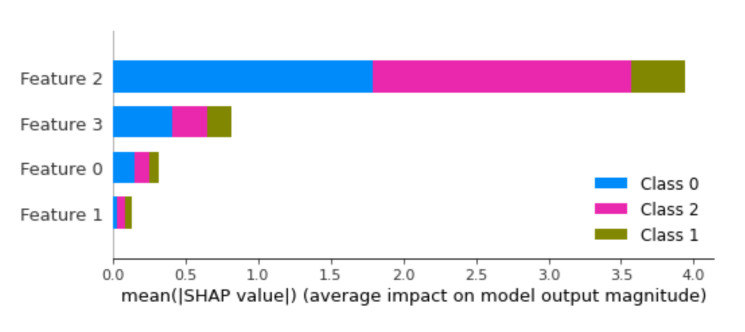
SHapley Additive exPlanations (SHAP) analysis results for the feature based classification method used in workflow 2.

Figure 9 presents the SHAP analysis results when Day 2 feature based CatBoost classifier training is considered. We can observe that $f_{2}$ feature components (minimum values) make the biggest contribution to the classification and that the $f_{3}$ feature components (maximum values) make the second largest contribution; the least contribution comes from the $f_{1}$ feature components (mean values). From this analysis, we conclude that we could slightly speed up the second workflow process by only extracting three feature components ($f_{2}$, $f_{3}$, and $f_{0}$) and training the classifier only with them.

### 6.4. Analysis of the Noise Artifacts

Day 1 recordings were acquired when it was very hot weather outside and the sun was shining straight through the meeting table from the large office windows (this information was provided by our industrial partner who collected the data in a real office environment). During Day 2 data acquisition, it was rather cloudy and the meeting room was not exposed to the sun rays. We have observed the effects of the outside weather conditions in our Day 1 data set as noise artifacts. We have also achieved less performance on the Day 1 recordings than the Day 2 recordings with both two workflows. It has been obvious that the outside weather impact on the meeting room has caused noise artifacts in the data set.

**Figure 10:**
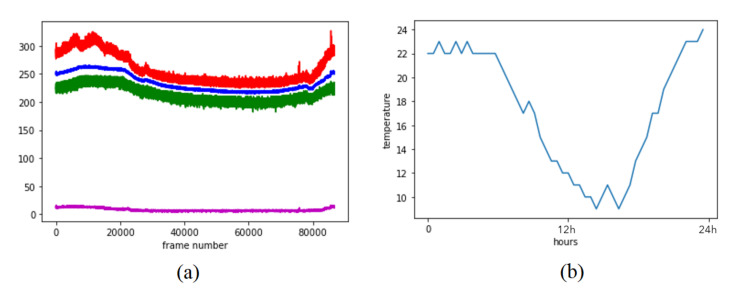
(**a**) Red; $f_{3}$, maximum pixel value of each frame (the maximum value is around 360, which corresponds to 36 degree Celsius). Blue; $f_{1}$ mean pixel values. Green; $f_{2}$ minimum pixel values. Magenta; $f_{0}$ standard deviation of the pixel values in each frame. (**b**) Outside temperature in Malmö, south Sweden, from the beginning to the end of the heat sensor recording used in (**a**).

The Day 3 recordings were captured in a 24 h time period and, thus, they contain the largest outside weather condition variety. Figure 10a illustrates the $f_{i}$ feature components for each frame. Figure 10b provides the plot of the outside temperature value in Malmö, Sweden—where the office is located—from the beginning to the end of the data acquisition process on the same date [32]. We notice a high correlation between the outside temperature and the $f_{1}$, $f_{2}$, and $f_{3}$ feature components (mean, minimum value, and maximum value, as seen with the blue, green and red colors, respectively). However, the $f_{0}$ feature component (standard deviation of each frame seen in the magenta color plot) looks just slightly affected by the outside temperature changes. Because all four features are affected in a similar direction (even though the $f_{0}$ feature component is affected less), we believe that the second workflow can do feature classification robustly, regardless of the outside temperature changes. However, the first workflow requires background correction using the mean values that were collected from the empty office. If the outside temperature changes a lot, this means that those values need to be re-calculated for the empty office again. When we look closely at Figure 10a, we notice two spikes (plotted in red) at the final frames of the recording. Tiny spikes on the same frames are also visible on the standard deviation values (plotted in magenta). When we look at our RGB camera recording that we have left in the office for validation (not for computing any results, only for calculating the performances of our experiments), we notice that one person has visited the meeting room at the time when the first spike occurred. Another two people had sat on the meeting table at the time when the second spike occurred. When we used the classifier (trained on Day 2 data) on these frames, once again we were able to predict the occupancy correctly. We also have correct occupancy predictions on samples of the empty office recordings of the different time frames of the 24 h recording. These results prove the robustness of the algorithm regarding the environmental noise artifacts.

We conclude that the first workflow (computer vision-based) is more sensitive to the environmental heat noise (changing outside temperature and the hitting sun rays) than the second workflow (feature classification-based). When looking at the empty office mean and standard deviations before and after the meeting in Day 1 (see Figure 5), we can see that the heat prints that are left from people sitting for a long time highly affect the standard deviation calculations. Therefore, the second workflow (machine learning-based) is likely to be less sensitive to the environmental heat noise, but more sensitive to the human heat print that is related noise artifacts.

### 6.5. Analysis of the Local Pixel Contributions

**Figure 11:**
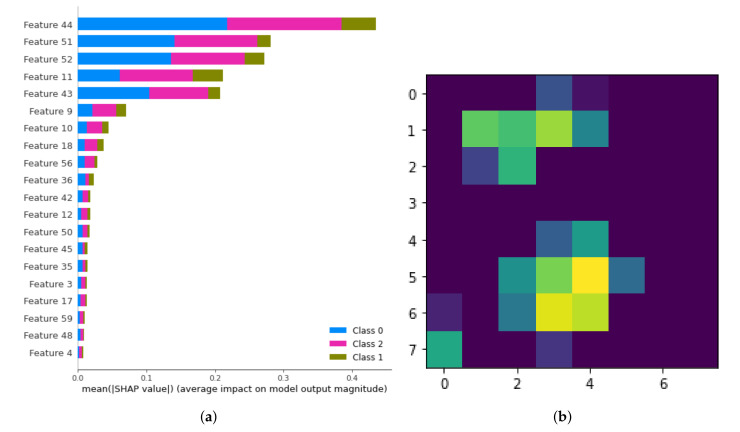
(**a**) Local contributions of pixels for performing good classifications considering heat recordings as time series for input. (**b**) Local contributions of each pixel are highlighted based on the explanations given in (**a**). Brighter pixels contribute more to the classification outcomes.

We developed a method inspired by LIME (Local Interpretable Model-agnostic Explanations) by Ribeiro et al. for explaining the local contributions of each pixel to the classification results [33]. LIME provides local explanations that can help to understand not only the trained model but also the contribution of each pixel for training such model. Thus, analyzing the pixel contributions provided by LIME can help us to understand whether the model is making decisions by considering the important locations in the image or not. If the model is making decisions by looking at pixels that are not very interesting, then we can assume that it would yield untrustworthy predictions. In such scenario, the pixels that do not make a valuable contribution to the classification can be removed and the classifier could be re-trained by using the pixels that are likely to hold more descriptive information to identify the occupancy of the scene. This pixel contribution analysis supports and complements the previous analysis carried out using SHAP. However, instead of looking at the contribution of each feature, we look at the contribution of each pixel to the classification and see whether we can trust our model’s decision making process or not. For this pixel analysis, we used again SHAP; however, we have now searched for the contribution of each pixel to the classification performance of the model which was trained with the Day 2 data. See illustration in Figure 11. In order to be able to do this feature analysis, we have considered each frame as a 1×64 size vector (since each frame has the size of 8×8). We named each component (each pixel) of this 1×64 size vector as Feature0,Feature2,…,Feature63 where Feature0 corresponds to the top left pixel and the rest of the pixels are named in a sequence of left to right for each row. Figure 11a shows the SHAP analysis results illustrating the importance of each pixel for the training process. The results show that Feature44 has made the biggest contribution for training the classifier, after that then Feature51,52,11,43,… show the larger contribution to train the classifier. When we find these components (which were named from 0 to 63) back in the 8×8 image, we can see which pixels were contributing to the classification and to which degree and which pixels were not contributing. Figure 11b show the contribution of each pixel with color codes. Here, brighter pixels correspond to the ones whose $f_{1,2,3,4}$ features helped most to train the classifier. We see that two seat positions are highlighted. We can verify that this is correct because features coming from seat positions helped to train the classifier to predict the occupancy. Therefore, the mapping that we see in Figure 11b shows us that our classifier is trustworthy. Unfortunately, at the left bottom pixel, we also see a highlight. We are aware that there is no seat at that location and we notice this highlighted pixel as noise in some of the Day 2 recordings. The results clarify that this noisy pixel also contributed to training the classifier. We learnt that it might be a good idea to block the features coming from this pixel before re-training the classifier.

## 7. Discussion

Herein, we will address our research questions and discuss the answers that we have found with our experiments. We also highlight the lessons that were learned by our empirical evaluations and the application of the explainable methods.

### 7.1. Can We Rely on Computer Vision or Machine Learning Based Methods in Order to Provide Robust Occupancy Prediction Solutions When We Use a Low-Cost and Low-Resolution Sensor?

We answer this question by offering two different workflows in our study. We saw that, while using a traditional computer vision method, we can offer a robust real-time solution. However, the computer vision based method that we designed works when an empty room recording is available. When it is not possible to find such recording, our feature classification based second workflow can provide reliable results in order to identify the number of people within the scene.

### 7.2. What Is the Best Occupancy Prediction Accuracy that We Can Achieve When Computer Vision Based or Machine Learning Based Methods Are Used?

We have reached over 80% occupancy prediction performance with the computer vision based workflow and over 90% performance with the feature classification based workflow. Choosing the right workflow for the specific use case will lead to the best performance. This method requires less noisy data, even though good occupancy prediction performances can be achieved by using the feature classification method. However, the computer vision based method is able to compensate for the noise artifacts of the environment (heated objects) and also the heat prints of people. For instance, if it is possible to record an empty office (without human heat prints), before each meeting, we could provide reliable performances with the computer vision based method. Feature based algorithm is recommended if empty office recordings are not available.

### 7.3. What Are the Sources of the Noise Artifacts and What Are the Possible Noise Compensation Methods to Overcome Errors Caused by Them?

We have noticed two significant noise factors that cause artifacts in our data. One is caused by the sun rays directly hitting the furniture and the objects in the office. Highly heated surfaces appear almost like a person and cause confusion to our algorithms. The second noise factor is the heat prints of people who used the room for a while (sitting at a meeting chair for half an hour, for instance). We saw that our first (computer vision based) workflow is more robust to this second kind of noise factor. However, we believe that using activity recognition methods in time series of heat recordings could increase the occupancy prediction accuracy. A time series analysis approach might also allow for identifying heat prints that become cooler in time (like a chair where the heat print might be lost slowly).

### 7.4. Comparison of the Two Different Workflows

Table 4 lists a comparison between the two proposed workflows, according to the results of our experiments. The table highlights the advantages and disadvantages of each workflow.

### 7.5. Further Recommendations

Looking at the results that are provided in Figure 4, we can make the following two suggestions in order to achieve good accuracy values when using the first workflow (computer vision-based):

- for the background analysis, m(x,y) and s(x,y) values should be calculated when the office has been empty for a while (to make sure that there are no heat prints from people); and,
- we recommend to re-calculate m(x,y) and s(x,y) when the outside heat is significantly different or the sun rays are illuminating more/less powerfully than the earlier empty room recordings.

When the second workflow is used, we recommend training the classifier when the noise artifacts are low (no heat prints from people who left a seat and no heavy sun light exposure on the objects). If the temperatures are varying significantly during the day, we recommend training the classifier with data from different times of the day.

## 8. Future Work

Algorithm-wise, we would like to focus on trying our solutions with multiple sensor data. In this case, our current efforts include: optimization of the sensor distribution in the environment, synchronization of the input data, overcoming challenges that come from the overlapping views, and providing results that are based on the fused information. Secondly, we will focus on developing new algorithms for identifying human activity in our low-resolution sensor recordings. We are currently working on a solution that is based on the use of a deep neural network, specifically Mask R-CNN [34]. We have conducted some early experiments training a Mask R-CNN network, which can learn identifying person positions from low-resolution heat sensor images.

Application-wise, we are interested in bringing these smart office solutions to other fields, e.g., elderly care environments. The rapidly growing old age population in many countries has increased the research that addresses challenges in Ambient Assisted Living. Robust smart office solutions, like those that are presented here, can be adopted in this area [35].

When considering both perspectives, algorithm and application-wise, we are interested in further developments towards the observation and analysis of very large areas (i.e., conference rooms, auditoriums, large hallways). We are currently working on finding optimal positions to capture recordings of a large area using multiple sensors.

## 9. Conclusions

Herein, we have proposed two new algorithm workflows for real-time occupancy prediction in smart office spaces while using very low-resolution heat sensor data that take the background noise generated by the surroundings into account. The first workflow is based on computer vision algorithms, while the second one, on feature extraction and machine learning. We have conducted experiments in order to answer three major research questions that are related to finding reliable prediction methods, which accuracy can be achieved and the identification of noise artifacts in the environment. Additionally, we have used state-of-the-art explainability methods to understand how various parameters affect the performance of the algorithms. We believe that the proposed workflows can be used in many application areas that are related to the automation and efficient use of offices, spaces, and buildings, in order to improve human well-being and the efficient and sustainable use of the environment. The analysis and methods employed and developed to understand the parameters and the environmental noise effects on the occupancy prediction problem might help other researchers to develop robust algorithms for processing very low-resolution sensor data.

## Figures and Tables

**Table 1:** Presence sensor recordings used for testing our algorithms.

Data Label	Description	Total People	Total Frames
$h_{1}\left(x,y\right)$	Day 1 before meeting	0	94
$h_{2}\left(x,y\right)$	Day 1 after meeting	0	45
$h_{3}\left(x,y\right)$	Day 1 present at 1	1	24
$h_{4}\left(x,y\right)$	Day 1 present at 1 2	2	30
$h_{5}\left(x,y\right)$	Day 1 present at 1 2 3	3	27
$h_{6}\left(x,y\right)$	Day 1 present at 1 2 3 4	4	51
$h_{7}\left(x,y\right)$	Day 1 present at 2 3 4	3	29
$h_{8}\left(x,y\right)$	Day 1 present at 3 4	2	36
$h_{9}\left(x,y\right)$	Day 1 present at 4	1	35
$h_{10}\left(x,y\right)$	Day 2 before meeting	0	94
$h_{11}\left(x,y\right)$	Day 2 present at 1 2	2	56
$h_{12}\left(x,y\right)$	Day 2 present at 2 3	2	47
$h_{13}\left(x,y\right)$	Day 2 present at 3	1	28
$h_{14}\left(x,y\right)$	Day 2 present at 3 4	2	31
$h_{15}\left(x,y\right)$	Day 3 24 h	max 2	661

**Table 2:** Performance of the computer vision based method on each recording which includes at least one person in the scene.

Data Label	Total People	Total Frames	TD (%)	FP (%)	FN (%)
$h_{3}\left(x,y\right)$	1	24	100	100	0
$h_{4}\left(x,y\right)$	2	30	100	86.60	0
$h_{5}\left(x,y\right)$	3	27	85.19	37.03	14.81
$h_{6}\left(x,y\right)$	4	51	94.11	0	5.89
$h_{7}\left(x,y\right)$	3	29	97.50	0	2.50
$h_{8}\left(x,y\right)$	2	36	80.55	2.77	19.45
$h_{9}\left(x,y\right)$	1	35	100	80	0
$h_{11}\left(x,y\right)$	2	56	100	0	0
$h_{12}\left(x,y\right)$	2	47	100	0	0
$h_{13}\left(x,y\right)$	1	28	100	0	0
$h_{14}\left(x,y\right)$	2	31	83.8	12	3.2

**Table 3:** Performance of the feature classification based method where the training and test sets are also listed.

Trained on 70% of	Tested on 30% of	TD (%)	FP (%)	FN (%)
Day 2	Day 2	97.64	0	2.36
Day 1	Day 1	95.48	27.60	4.52
Day 1	Day 2	94.70	0	5.30
[Day 1, Day 2]	Day 1	96.70	30.00	3.30

**Table 4:** Comparison of the different occupancy prediction workflows (one computer vision-based, two machine learning-based.

Condition	Workflow 1	Workflow 2
Dependency on an empty room recording	Good performances obtained only by having an empty room recording before meetings	It is not necessary to have an empty room recording
Dependency on a training data set	The algorithm only needs an empty room recording as a condition. Other, training data is not necessary.	The algorithm needs a training data set which includes recordings with different numbers of people in the scene.
Dependency on the environmental noise (i.e., sun exposure)	The algorithm behaves robustly when a similar environment is recorded when the office is empty. Otherwise, it is prone to provide false positives.	If the algorithm has such examples in the training data set, it provides reliable results.
Dependency on human heat prints	The algorithm behaves robustly, since such prints can be eliminated with the background compensation module.	The algorithm cannot easily distinguish human heat prints. The algorithm is prone to provide false positives.
Dependency on other heated objects (i.e., computer, hot drinks, etc.)	The algorithm behaves robustly, because of the position of our sensor (shown in Figure 2), such objects appear too small in the images and they do not contribute as a noise factor.	Same as in Workflow 1.

## Abbreviations

The following abbreviations are used in this manuscript:

AI	Artificial Intelligence
CNN	Convolutional Neural Network
FN	False Negative
FP	False Positive
IoT	Internet of Things
LIME	Local Interpretable Model-agnostic Explanations
LSTM	Long Short Term Memory
ML	Machine Learning
PIR	Passive Infrared
SHAP	SHapley Additive exPlanations
TD	True Detection
XAI	eXplainable Artificial Intelligence
